# MLV requires Tap/NXF1-dependent pathway to export its unspliced RNA to the cytoplasm and to express both spliced and unspliced RNAs

**DOI:** 10.1186/1742-4690-11-21

**Published:** 2014-03-05

**Authors:** Lucie Pessel-Vivares, Mireia Ferrer, Sébastien Lainé, Marylène Mougel

**Affiliations:** 1 UMR5236 CNRS, UM1, UM2, CPBS, 1919 Route de Mende, Montpellier, France

**Keywords:** Retrovirus, Tap/NXF1, RNA, Nuclear export, MLV, Intron-containing RNA, Splicing, Fluorescence microscopy, Quantitative RT-PCR, RIP assay

## Abstract

**Background:**

Eukaryotic cells have evolved stringent proofreading mechanisms to ensure that intron-containing mRNAs do not leave the nucleus. However, all retroviruses must bypass this checkpoint for replication. Indeed, their primary polycistronic transcript (Full-Length) must reach the cytoplasm to be either translated or packaged as genomic RNA in progeny viruses.

Murine leukemia virus (MLV) is a prototype of simple retroviruses with only two well-regulated splicing events that directly influence viral leukemogenic properties in mice. Several *cis*-elements have been identified in the FL RNA that regulate its cytoplasmic accumulation. However, their connection with an export mechanism is yet unknown. Our goal was to identify the cellular pathway used by MLV to export its RNAs into the cytoplasm of the host cells.

**Results:**

Since other retroviruses use the CRM1 and/or the Tap/NXF1 pathways to export their unspliced RNA from the nucleus, we investigated the role of these two pathways in MLV replication by using specific inhibitors. The effects of export inhibition on MLV protein synthesis, RNA levels and RNA localization were studied by Western blotting, RT-qPCR, fluorescence microscopy and ribonucleoprotein immunoprecipitation assays. Taken together, our results show for the first time that MLV requires the Tap/NXF1-mediated export pathway, and not the CRM1 pathway, for the expression of its spliced and unspliced RNAs and for FL RNA nuclear export.

**Conclusions:**

By contrast to HIV-1, MLV recruits the same pathway for the cytoplasmic expression of its spliced and unspliced RNAs. Thus, MLV RNA expression depends upon coordinated splicing/export processes. In addition, FL RNA translation relies on Tap/NXF1-dependent export, raising the critical question of whether the pool of FL RNA to be packaged is also exported by Tap/NXF1.

## Findings

Cellular mRNAs are fully spliced prior to their export from the nucleus. The quality of gene expression is assured by proofreading mechanisms that eliminate unprocessed or irregular pre-mRNAs [1,2]. Retroviral RNA, however, needs to be exported to the cytoplasm in a partially spliced or totally unspliced, full-length (FL), form in order to serve as a template for protein synthesis. Furthermore, in addition to producing the structural proteins (Gag) and enzymes (GagPol), unspliced RNA also acts as genomic RNA to be packaged into virions. To achieve this nuclear export of incompletely spliced and FL RNAs, complex retroviruses, such as HIV, encode an adaptor protein (Rev) that bridges the FL RNA, via its Rev-responsive element (RRE), and the CRM1 nuclear export factor [3]. Simpler retroviruses such as Mason-Pfizer monkey virus (MPMV), recruit the global mRNA export pathway mediated by the cellular Tap factor (also called NXF1) that directly binds a constitutive transport element (CTE) in the FL RNA [4]. The scenario appears to be more complex for the export of avian retroviral FL RNA. Indeed, Rous sarcoma virus (RSV), another simple retrovirus, relies on the CRM1 pathway using Gag protein as an adapter [5] and on the Tap pathway *via* two direct repeat (DR) sequences in the FL RNA [6].

The murine leukemia virus (MLV) was among the first retroviruses to be studied and constitutes the prototype for simple retroviruses. Moloney-MLV, in particular, has been key in our understanding of basic cellular processes such as the discovery of oncogenes [7], eukaryotic gene regulation, viral pathogenesis [8], and therapeutic gene transfer trials with MLV-based vectors [9]. Although MLV has been extensively studied, the export pathway used by MLV RNAs to reach the cytoplasm remains unidentified. Several *cis*-elements, such as the Psi motifs and R region sequences in the 5' UTR of FL RNA, have been reported to promote the cytoplasmic accumulation of FL RNA [10-13]. However, the fact that these *cis*-acting elements also modulate RNA splicing efficiency, RNA stability and virus assembly has made it difficult to elucidate the nuclear export pathway that they use (reviewed in [14]).

In this study, we have investigated the nuclear export of MLV RNAs. We show that inhibition of the Tap-pathway dramatically decreases viral protein production and simultaneously decreases the levels of spliced and unspliced MLV RNAs. A sensitive and specific fluorescence detection method was used to study the export of unspliced viral RNA. RNA imaging and ribonucleoprotein (RNP) immunoprecipitation assays (RIP) provided strong evidence for the recruitment of the Tap-pathway by MLV to export its RNAs to the cytoplasm.

### MLV expression is dependent on the Tap pathway

**Figure 1:**
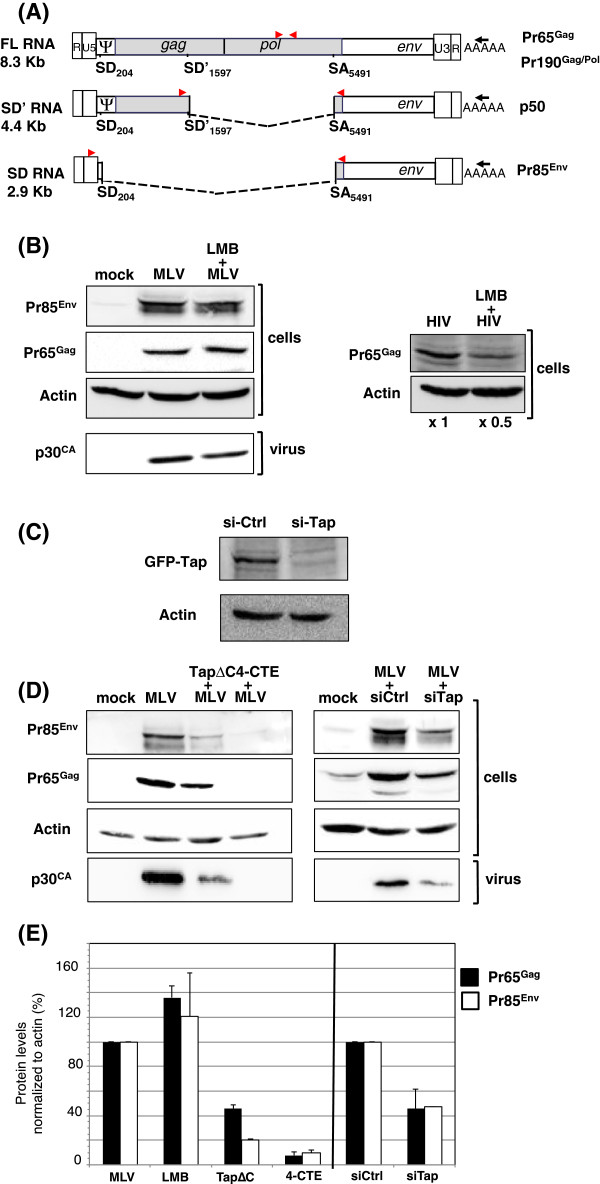
**Effects of CRM1 and Tap inhibition on MLV expression. (A)** RNAs produced by MLV and their encoded proteins. Black arrows correspond to the RT primers while positions of the RT-qPCR primers used to amplify the different RNAs are indicated by red arrows. **(B)** Investigation of the CRM1 pathway by Western blot analysis of MLV or HIV proteins produced in LMB-treated NIH3T3 or 293 T cells, respectively. Cells were transfected with 2 μg of pMov9.1 or pNL4.3 plasmid, the molecular clones of Mo-MLV and HIV-1 respectively, for 48 h and incubated for the last 8 h with 20 nM LMB (LC Laboratories) before harvesting. **(C)** Activity of the Tap siRNA. 1 μg of TAPΔC was cotransfected in NIH3T3 cells with 100 pmol of Tap Stealth RNAi (invitrogen) or control siRNA (Eurogentec). Cell were collected 48 h p.t. and Tap expression analyzed by Western blotting with anti-GFP antibodies (Roche). **(D)** Investigation of the Tap pathway by Western blot analysis of MLV proteins produced in NIH3T3 cells transfected for 48 h with 1 μg pMov9.1 with either 1 μg of peGFP-TapΔC [19], 1.5 μg of p3-CCCC (4-CTE) [18] or 1.5 μg of empty pcDNA3.1 vector. In all experiments, MLV:inhibitor ratios are 1:3 for peGFP-TapΔC and p3-CCCC (or pcDNA3.1). For RNA interference, NIH3T3 cells were co-transfected for 48 h with 2 μg pMov9.1 and 100 pmol of either Tap stealth RNAi as described in [18], or control siRNA. Viruses were collected by ultracentrifugation from culture supernatant and loaded on SDS-PAGE. Procedure and antibodies used to detect viral proteins are described in [16]. Actin was detected with a rabbit polyclonal antibody (1/300) (Sigma) and a peroxidase-conjugated (HRP) goat anti-rabbit antibody (1:4000) (Sigma). **(E)** Band integrated densities were determined with the ImageJ software and normalized to actin. Intensities are presented as mean +/− SEM.

MLV expression relies on the production of three RNA species (Figure 1A): i) the primary transcript, an 8.3 kb unspliced RNA (FL) encoding for Gag (Pr65^Gag^) and GagPol (Pr190^Gag/Pol^) precursors; ii) a partially-spliced 4.4 kb RNA (SD' or SD'^p50^) encoding for the p50 protein whose function remains unknown [15], and iii) a fully-spliced 2.9 kb mRNA (SD or SD^Env^) encoding for the envelope (Pr85^Env^). How these spliced and unspliced MLV RNAs exit the nucleus to be translated in the cytoplasm is unknown. Since other retroviruses use the CRM1 and/or Tap pathways to export their unspliced RNA, we investigated these two pathways. First, murine NIH3T3 cells transfected with the Moloney-MLV molecular clone (pMov9.1) were treated with the CRM1 inhibitor leptomycin B (LMB) and MLV expression was monitored by Western blot analysis of Pr85^Env^ and Pr65^Gag^ proteins translated from spliced and unspliced RNAs, respectively. LMB treatment impaired neither spliced or unspliced MLV RNA expression in NIH3T3 cells while it reduced HIV FL RNA expression in 293 T cells as expected [16] (Figure 1B). Then, to examine the Tap pathway we used three different strategies to specifically block this pathway in NIH3T3 cells: i) RNA-induced silencing (Tap siRNA) to downregulate Tap expression (Figure 1C) [17], ii) over-expression of an RNA competitor harboring four copies of MPMV CTE (4-CTE) that interacts with the Tap protein [18] and iii) expression of a Tap mutant lacking the C-terminal residues 518 to 619 (TapΔC) that still binds mRNA but blocks its export in a dominant-negative fashion [19]. Indeed, the C-terminal domain of Tap is essential for transferring the RNA cargo through the nuclear pore complex. Expressing TapΔC reduced by 50% and 80% the Pr65^Gag^ and Pr85^Env^ protein levels respectively, while the Tap siRNA reduced both protein levels to a similar extent (60%). Remarkably, MLV expression was abolished by the 4-CTE competitor (Figures 1D-E). All of these effects caused subsequent severe defects in virus production (Figure 1D, lower panel). These results reveal that expression of spliced SD and FL RNAs were both dependent upon the Tap pathway which probably promotes RNA nuclear export and subsequent cytoplasmic expression.

### Tap-pathway inhibition decreases the spliced and unspliced MLV RNA levels

**Figure 2:**
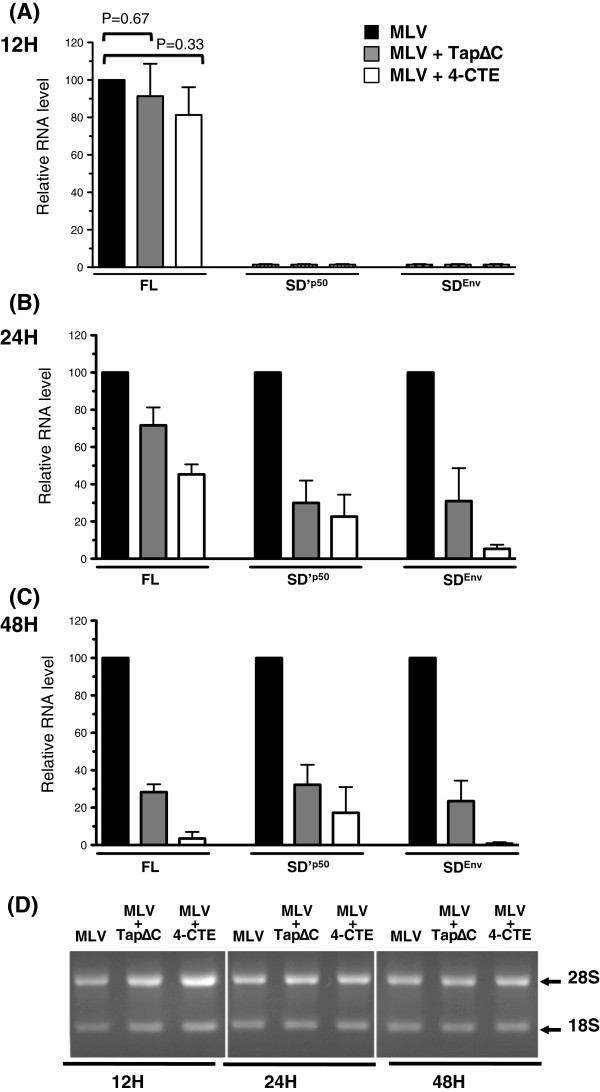
**Effects of Tap inhibitors on MLV RNA levels.** Transfections were performed with pMov9.1Δenv, a Mo-MLV molecular clone deleted of the HpaI segment (1.38Kb) in the *env* gene in order to prevent re-infection events and the inhibition conditions were as in Figure 1. At 12 h **(A)**, 24 h **(B)** and 48 h **(C)**, RNAs were extracted, DNAse treated, and analyzed by RT-qPCR with an MLV standard curve as described in [15]. Primers used are indicated in Figure 1A and their sequences are given in Additional file 1 Table S1. The RT reaction was performed using oligodT or specific antisense primer and followed by different specific qPCR amplifications. Controls were systematically performed to check for DNA contamination by running an RT reaction without enzyme. Background RNA copies measured in untransfected cells were removed from values and then normalized to the GAPDH mRNA control monitored with specific probes as in [16]. Real time PCR was performed with the FastStart SYBRGreen (Roche) on a RotorGene (Corbett Research). Ribosomal RNAs were loaded on agarose gel and detected with SybrGreen staining as controls **(D)**.

To further examine the effects of TapΔC and 4-CTE on MLV expression, we undertook a quantitative analysis of the viral RNAs at different time points. NIH3T3 cells were transfected with an *env*-deleted MLV in order to prevent re-infection events. Total cellular RNAs were extracted at 12, 24 and 48 h post-transfection (p.t) and MLV RNAs were specifically quantitated by RT-qPCR, as previously described [15] (Additional file 1: Table S1). At 12 h p.t., the FL RNA level remained stable in the presence of TapΔC or 4-CTE while the spliced SD'^P50^ and SD^Env^ remained at undetectable levels, probably due to incomplete splicing (Figure 2A). Decreased amounts of the three RNA species were observed at 24 h p.t. (Figure 2B) and their amounts were further decreased at 48 h (Figure 2C), indicating that all spliced and unspliced RNAs were affected by the Tap inhibition. As observed for the proteins (Figure 1), 4-CTE induced stronger effects on RNA than the TapΔC mutant. A relative correlation was observed between the FL and SD^Env^ RNA levels and the protein production. As a control, ribosomal RNAs, that are exported by the CRM1 pathway, were insensitive to the Tap inhibitors and remained stable over time, excluding the possibility of a global toxicity effect of Tap inhibition (Figure 2D). The decreased levels of FL RNA in cells observed at 48 h and 24 h p.t could be the result of its instability and/or increase in splicing. However, levels of spliced RNAs were also decreased when Tap was blocked. Therefore, our results support the notion that Tap inhibition induced the nuclear retention of the MLV spliced and unspliced RNAs and led *in fine* to their degradation by the cellular machinery, as reported for cellular mRNAs [1].

### TapΔC inhibits nuclear export of viral unspliced RNA

**Figure 3:**
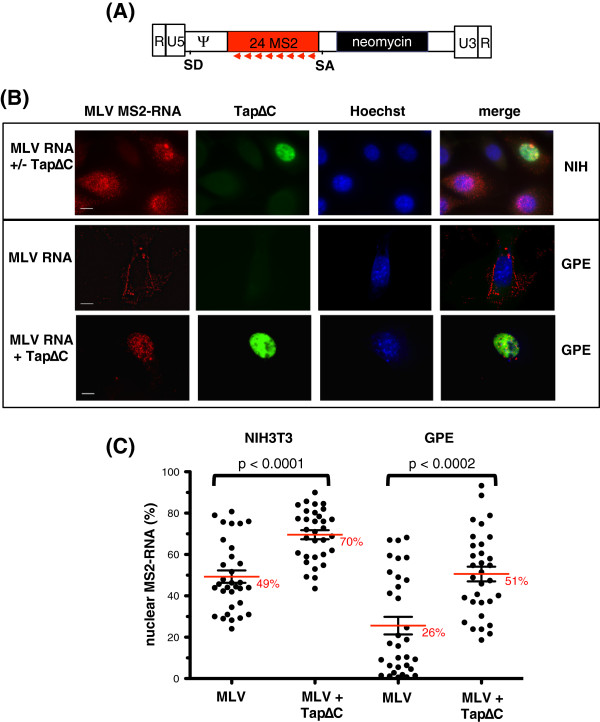
**Analysis of MLV export by fluorescence microscopy. (A)** Map of the reporter MS2- RNA. Red arrows correspond to the Cyan3-labelled probe used in FISH. **(B)** Reporter MS2-RNA was expressed in NIH3T3 or packaging GPE cell lines transfected or not with peGFP-TapΔC and fixed in 4% paraformaldehyde between 8-12 h p.t for FISH analysis using Cy3-MS2 probes as described [24]. Cells were imaged on a 100× NA 1.4 wide-field microscope (Zeiss AxioimagerZ1) equipped with a Coolsnap HQ2 camera. Stacks of images were recorded for Cy3 (MS2 RNA), GFP (TapΔC) and Hoechst (nucleus) fluorescence over the depth of the cell using a Z step of 0.3 μm, and represented as stacked images. Scale bar is 10 μm. All images were processed using the ImageJ software. **(C)** For quantitative analysis of viral RNA localization, filtered images (by subtraction of the blurred image using a sigma radius of 20) were stacked and automatically thresholded. Values for the integrated density (Mean Fluorescence intensity × Area corresponding to Cy3 signal) in the different regions of interest, nucleus and cytoplasm, were calculated using the Particle Analysis plugin in ImageJ. Data are expressed as the percentage of nuclear signal, with the mean +/− SEM values of 32 cells for each condition.

To investigate the requirement of Tap for MLV RNA export, we used a fluorescence *in situ* hybridization approach (FISH) [20] combined to MS2-RNA tagging that provides the high sensitivity required for unspliced viral RNA detection at a very short time after transfection, when RNA splicing is still incomplete and degradation of unexported RNA molecules not yet activated (Figure 2). Previous studies have shown that an MLV reporter RNA (PINA10Psiwt) sharing LTR, promoter, functional splice sites and Psi signal with the FL RNA, can mimic the intracellular transport of the FL RNA [10] and can be ultimately packaged into viral particles [21,22]. To achieve a high-resolution detection of single RNA molecules, 24-MS2 copies were inserted into the intron of this MLV reporter RNA (Figure 3A), which retains its packaging ability (data not shown) as previously reported [23]. First, the effects of TapΔC inhibition were examined in NIH3T3 cells and FISH was performed 8-12 h p.t with a probe carrying 4 cyan3 molecules and targeting the 24 MS2 sites [24]. RNA imaging revealed that GFP-TapΔC increased the nuclear level of unspliced MS2-RNA by 20%. Therefore, 70% of the viral RNA accumulated in the nucleus in the presence of Tap inhibitor (Figure 3B-C). Nuclear export was then investigated in a viral GPE packaging cell line, an NIH3T3 cell derivative. GPE provides MLV Gag and Env proteins expressed from two distinct cDNA plasmids and produces virus-like particles that can encapsidate the MS2-RNA reporter when expressed in *trans*. In this viral context, the reporter RNA localized mainly to the plasma membrane, with reduced nuclear localization. There were also numerous fluorescent puncta in the extracellular space that are probably associated with released viral particles. As in the NIH3T3 context, TapΔC induced a 20% increase of nuclear RNA retention. Taken together, these results establish that TapΔC blocked the nuclear export of the MLV reporter RNA, regardless of whether MLV proteins were present, and validates the requirement for the Tap-pathway to export MLV RNA from the nucleus.

### TapΔC interacts with spliced and unspliced MLV RNAs

**Figure 4:**
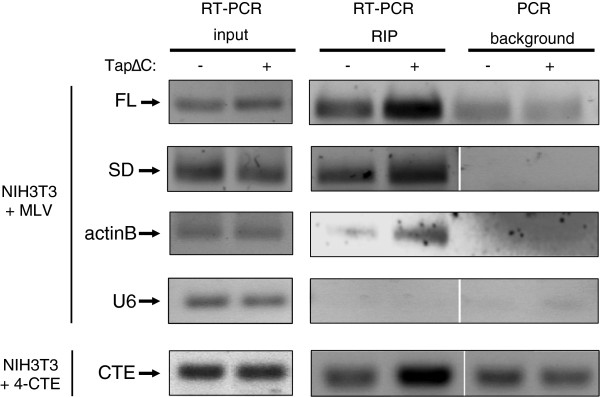
**Co-immunoprecipitation of spliced or unspliced MLV RNA with TapΔC factor.** NIH3T3 cells were cotransfected with 10 μg of peGFP-TAPΔC and 10 μg of pMov9.1 or 7 μg of peGFP-TAPΔC and 10,5 μg of p3-CCCC (4-CTE). At 24 h p.t., cells were extracted with 1 ml of lysis buffer (1% triton X-100, 150 mM NaCl, 50 mM Tris–HCl, complete anti-protease cocktail from Roche) and precleared with Protein A sepharose beads (invitrogen) during one hour at 4°C. 100 μl of cell lysates were saved to control transfection efficiencies by monitoring RNA levels (input). 900 μl of cell lysates were incubated 3 hours at 4°C with beads that specifically bind GFP (GFP-Trap® Chromotek) in order to co-immunoprecipitate GFP-TapΔC/RNA complexes. After disruption of the RNP complexes, RT-PCRs were performed on the precipitated RNA and the input to specifically amplify MLV RNA, or 4-CTE RNA, and two cellular RNA controls: actinB mRNA and U6 snRNA, the nuclear export of which rely on the Tap and CRM1 pathways, respectively. Control PCR experiments were systematically performed without prior RT reaction as a control for DNA contamination of RNA extracts. Amplicons were analyzed on 1% agarose gel.

To directly address the recruitment of the Tap factor by MLV, we performed RIP assays to isolate RNA-TapΔC complexes. NIH3T3 cells were transfected with the MLV molecular clone with or without the peGFP-TapΔC plasmid, and cell lysates were incubated with GFP-TRAP beads (Chromotek). MLV RNAs retained by TapΔC on beads were amplified by RT-PCR specific to FL or spliced (SD^Env^) RNA (as in Figure 2) and corresponded to a stronger band on agarose gel compared to the signal observed in the absence of Tap (Figure 4). Control PCR amplifications were ran without prior RT reaction to monitor DNA contamination. Similarly, we measured the levels of cellular U6 snRNA (negative control) and transfected 4-CTE and cellular actinB mRNA (positive controls), in immunoprecipitates. As expected, actinB and the 4-CTE mRNAs, both of which are exported by the Tap pathway, interacted with TapΔC. In contrast, U6 snRNA, which is exported via the CRM1 protein in mouse fibroblasts [10,25] did not, thus validating the specificity of the RIP assays (Figure 4). These results demonstrate the physical interaction between unspliced or spliced MLV RNA with TapΔC. However, it cannot be excluded that a cellular or viral cofactor mediates this interaction.

In this report, different strategies were used to block the Tap-pathway, in *cis* with siRNA or excess of 4-CTE RNA competitor and in *trans* with a dominant-negative Tap mutant (TapΔC). We have demonstrated for the first time that Tap is required for MLV expression and that it interacts with both spliced and unspliced MLV RNAs. When interacting with TapΔC, the unspliced RNA was restricted to the nucleus, indicating that MLV RNA export requires the Tap-dependent pathway. Our results support the notion that MLV recruits the same pathway to export its spliced and unspliced RNAs. In contrast to HIV, which uses different export pathways to transport its fully-spliced and unspliced RNAs, MLV regulates the expression of its FL RNA through a highly coordinated splicing/export process. Furthermore, the Tap-pathway is a prerequisite to the translation of the two MLV RNA species, since Gag and Env protein levels were sensitive to TapΔC. Unlike HIV-1, MLV has two distinct pools of FL RNA with two different destinies: translation and packaging into new particles [26]. In this scenario, our results support the notion that the translated RNA pool is exported by the Tap pathway. Whether the packageable FL RNA is exported by the same Tap mechanism remains to be established although this will be difficult to do. It will be interesting to determine whether MLV uses a similar replication strategy to RSV, another simple retrovirus. Interestingly, it has been postulated that RSV, after exporting a fraction of FL RNA from the nucleus to produce large amounts of structural Gag proteins for virus formation, uses excess Gag proteins to fetch the remainder of the FL RNA in the nucleus and to route it to the virus assembly sites where it serves as genome in new virus particles. Complex retroviruses regulate this temporal switch between early and late steps of replication differently, by using early and late gene expression (for review [27]). It must now be established whether nuclear export pathway use regulates cytosolic RNA fate.

## Competing interests

The authors declare that they have no competing interests.

## Authors’ contributions

SL and MM designed and analyzed the research; LPV, SL, and MFA performed research, collected data, and helped in finalizing the manuscript. MM wrote the manuscript. All authors read and approved the final manuscript.

## Supplementary Material

Additional file 1: Table S1 The following oligonucleotides were used for the RT-PCR assays. The name refers to the RNA target. Click here for file
