# Supplementary material for: MLV requires Tap/NXF1-dependent pathway to export its unspliced RNA to the cytoplasm and to express both spliced and unspliced RNAs
Source: Retrovirology. 2014 Mar 5;11:21. doi: 10.1186/1742-4690-11-21 (PMC4015919; doi:10.1186/1742-4690-11-21)
Supplement: Additional file 1: Table S1 — The following oligonucleotides were used for the RT-PCR assays. The name refers to the RNA target. [file 1742-4690-11-21-S1.pdf]

**Supplemental Table 1:** The following oligonucleotides were used for the RT-PCR assays. The name refers to the RNA target.

| Name | Sequence (5'--> 3') |
|---|---|
| FL sense | TATCGGGCCTCGGCAAGAAAG |
| FL antisense | AAACAGAGTCCCCGTTTTGGTG |
| SD sense | GTGGTCTCGCTGTTCCTTGGGA |
| SD' sense | CTGCTGACGGGAGAAGAAAAACA |
| SD-SD' antisense | GCGGACCCACACTGTGTC |
| U6 sense | GCTCGCTTCGGCAGCACATATACT |
| U6 antisense | TATGGAACGCTTCACGAATTTGCG |
| actinB sense | CCAACTGGGACGACATGGAGAAG |
| actinB antisense | CAGAGGCATACAGGGACAGCACAG |
